# Plyometrics Can Preserve Peak Power During 2 Months of Physical Inactivity: An RCT Including a One-Year Follow-Up

**DOI:** 10.3389/fphys.2018.00633

**Published:** 2018-05-29

**Authors:** Andreas Kramer, Jakob Kümmel, Albert Gollhofer, Gabriele Armbrecht, Ramona Ritzmann, Daniel Belavy, Dieter Felsenberg, Markus Gruber

**Affiliations:** ^1^Sensorimotor Performance Lab, University of Konstanz, Konstanz, Germany; ^2^Department of Sports Science, University of Freiburg, Freiburg, Germany; ^3^Center of Muscle and Bone Research, Charité Universitätsmedizin Berlin, Berlin, Germany; ^4^Institute for Physical Activity and Nutrition, School of Exercise and Nutrition Sciences, Deakin University, Geelong, VIC, Australia

**Keywords:** bed rest, specificity, SSC, exercise, countermeasure, power, rate of force development

## Abstract

**Objective:** Inactivity results in a marked loss of muscle function, especially in movements requiring high power, force, and rate of force development. The aim of the present study was to evaluate if jump training can prevent these deteriorating effects of physical inactivity.

**Methods:** Performance and muscle activity during several types of jumps was assessed directly before and after 60 days of bed rest as well as during follow-up visits in 23 male participants. Participants in the jump training group (JUMP, 12 participants) trained 5–6x per week during the bed rest period in a sledge jump system that allows jumps in a horizontal position, whereas the control group (CTRL, 11 participants) did not train.

**Results:** Performance and muscle activity considerably decreased after bed rest in the control group but not in the training group, neither for countermovement jumps (peak power CTRL −31%, JUMP +0%, group × time interaction effect *p* < 0.001), nor for squat jumps (peak power CTRL −35%, JUMP +1%, *p* < 0.001) and repetitive hops (peak force CTRL −35%, JUMP −2%, *p* < 0.001; rate of force development CTRL −53%, JUMP +4%, *p* < 0.001). The control group's performance had returned to baseline 3 months after bed rest.

**Conclusion:** Despite the short exercise duration, the jump training successfully prevented power and strength losses throughout 2 months of bed rest.Thus, plyometrics can be recommended as an effective and efficient type of exercise for sedentary populations, preventing the deterioration of neuromuscular performance during physical inactivity.

## Introduction

Physical performance decreases with age (Ali and Garcia, 2014) and disuse, for example during bed rest (Pavy-Le Traon et al., 2007) or exposure to weightlessness (Adams et al., 2003). Many aspects of physical performance are affected— such as strength and aerobic capacity—but peak power seems to be affected to a higher extent than for example maximal voluntary strength (Skelton et al., 1994; Alkner and Tesch, 2004).

To test potential countermeasures for the decline of neuromuscular performance, several bed rest studies enforcing strict physical inactivity have been conducted. Exercise and nutrition interventions that have been tested include protein supplementation, vibration training, and resistance strength training. However, none of these countermeasures could completely prevent the loss of peak power (Rittweger et al., 2007; Buehring et al., 2011; Gast et al., 2012). This is true even for countermeasures that were successful in maintaining maximal leg muscle strength, prompting the authors to conclude that preserving muscle mass and strength does not necessarily preserve muscle power, and that there is a need for training methods that maintain both strength and power (Buehring et al., 2011).

Moreover, muscle power and rate of force development correlate better with quality of life and activities of daily living than muscle mass or strength (Katula et al., 2008; Reid and Fielding, 2012), and seem to be key factors in preventing falls (Skelton et al., 2002; Bento et al., 2010; Tschopp et al., 2011). Consequently, sedentary and elderly populations would greatly benefit from an intervention that can effectively prevent the pronounced decline of power and rate of force development.

One type of exercise that has the potential to do so is jumping: it is the exercise mode with the highest power output (Davies, 1971), a high rate of force development, and has repeatedly been shown to increase leg muscle strength and power in healthy participants not subjected to bed rest (Saez-Saez de Villarreal et al., 2010; Marián et al., 2016). The aim of the present study was to assess if jump training can counteract the pronounced decline in leg muscle power, maximal force, and rate of force development during jumps caused by 2 months of strict bed rest. To render jumps in a supine position possible, a sledge jump system (SJS) was used that had already been successfully tested in previous studies (Kramer et al., 2010, 2012a,b). As results from a 90-day bed rest study showed that performance improvements can be specific to the device used during the training and may not transfer to standardized tests such as vertical jumps (Rittweger et al., 2007), a secondary goal of the present study was to examine if the training effects in the SJS transferred to normal jumps on the ground, and whether potential differences would be reflected in muscle activity. Previous studies have shown that reactive jumps such as drop jumps and hops require a high preactivation of the leg extensors (Gollhofer and Kyröläinen, 1991; Kramer et al., 2012b), whereas countermovement jumps exhibit low muscle activity during the eccentric phase and high activity during the concentric phase (Finni et al., 2000).

We hypothesized that the jump training would prevent the decline in power, force, and rate of force development caused by 2 months of bed rest, with significant group × time interaction effects, and that these group differences in neuromuscular performance would also be reflected in the muscle activity.

## Methods

### Study design

**Figure 1:**
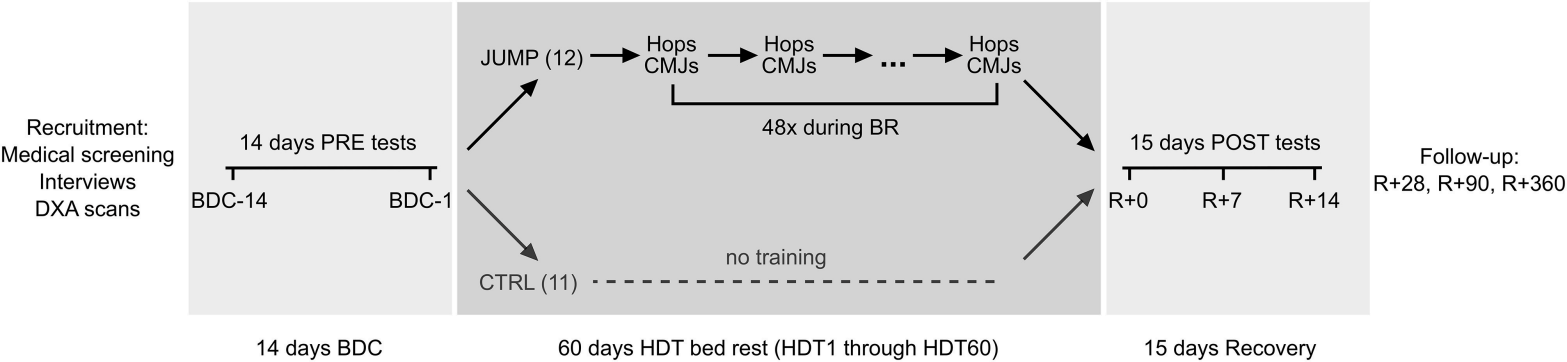
Study overview. Prior to the bed rest phase, participants spent 14 days in the bed rest facility for familiarization and baseline data collection (BDC-14 through BDC-1). In the morning of the first head-down tilt bed rest day (HDT1), participants were randomly assigned to either the training group (JUMP, total of 48 training sessions during the 60 days of bed rest) or the control group (CTRL). After the 60 days of HDT bed rest, participants were re-ambulated and stayed for an additional 15 days in the bed rest facility for measurements and recovery (R+0 through R+14). They returned to the bed rest facility on several occasions (R+28, R+90, and R+360) for follow-up measurements.

This single-center, parallel-group randomized controlled training study with balanced randomization was conducted at the German Aerospace Center (DLR) in Cologne. It consisted of 15 days of baseline data collection (BDC-15 through BDC-1), 60 days of strict 6° head-down tilt bed rest (HDT1 through HDT60) and 15 days of recovery (R+0 through R+14), see Figure 1. Details about the study can be found in Kramer et al. (2017b) and (Kramer et al., 2017a). During the bed rest period the subjects maintained the 6° HDT for 24 h/day. During the adaptation and recovery phases (BDC and R), physical activity was restricted to free movement within the ward and reeducation training during recovery. During the entire study, the subjects received a strictly controlled diet. After being released from the bed rest facility on day R+14, subjects returned on day R+28, R+90, and R+360 for follow-up measurements. Jump performance of all subjects was tested1 day after arrival at the facility (BDC-14), directly before (BDC-1), and after bed rest (R+0), as well as during recovery (R+7 and R+13) and follow-up (R+28, R+90, and R+360). In addition, the training group's jump performance was assessed on days HDT20 and HDT40 at the beginning of that day's training session. The primary endpoint with respect to the efficacy of the training intervention was peak power of the lower limbs, assessed during vertical jumps at R+0 compared to baseline at BDC-1. The study was registered with the German Clinical Trial Registry (DRKS, registration number DRKS00012946, 18th of September 2017).

### Subjects

Of the 24 healthy male subjects that were enrolled in the study, one subject discontinued the study on BDC-4 for medical reasons unrelated to the study. In the morning of the first bed rest day (HDT1), subjects were randomly allocated to either the jump training group (JUMP, 12 participants, age 30 ± 7 years, height 181 ± 7 cm and body mass 77 ± 7 kg) or the control group (CTRL, 11 participants, age 28 ± 6 years, height 181 ± 5 cm, and weight 76 ± 8 kg). Two of the 23 subjects that completed the study (one CTRL, one JUMP) were re-ambulated after, respectively, 49 and 50 instead of 60 days of HDT due to medical reasons, and completed the recovery and follow-up phase with the scheduled measurements. This study was carried out in accordance with the recommendations of the Ärztekammer Nordrhein. The protocol was approved by the ethics committee of the Northern Rhine Medical Association (Ärztekammer Nordrhein) in Duesseldorf, Germany, as well as the Federal Office for Radiation Protection (Bundesamt für Strahlenschutz). All subjects gave written informed consent in accordance with the Declaration of Helsinki.

Inclusion criteria were as follows: male, age between 20 and 45 years, body mass index between 20 and 26 kg/m2, non-smoking, no medication, no competitive athlete, and no history of bone fractures. Exclusion criteria were chronic hypertension, diabetes, obesity, arthritis, hyperlipidaemia, hepatic disease (A, C), disorder of calcium or bone metabolism, or heritable blood clotting disorders. Volunteers that were medically eligible for the study subsequently underwent psychological screening, involving questionnaires, and interviews. The recruitment process was concluded by a dual energy X-ray absorptiometry (DXA) screening of the bone mineral density of the femur and the lumbar vertebra column.

### Training device and familiarization

**Figure 2:**
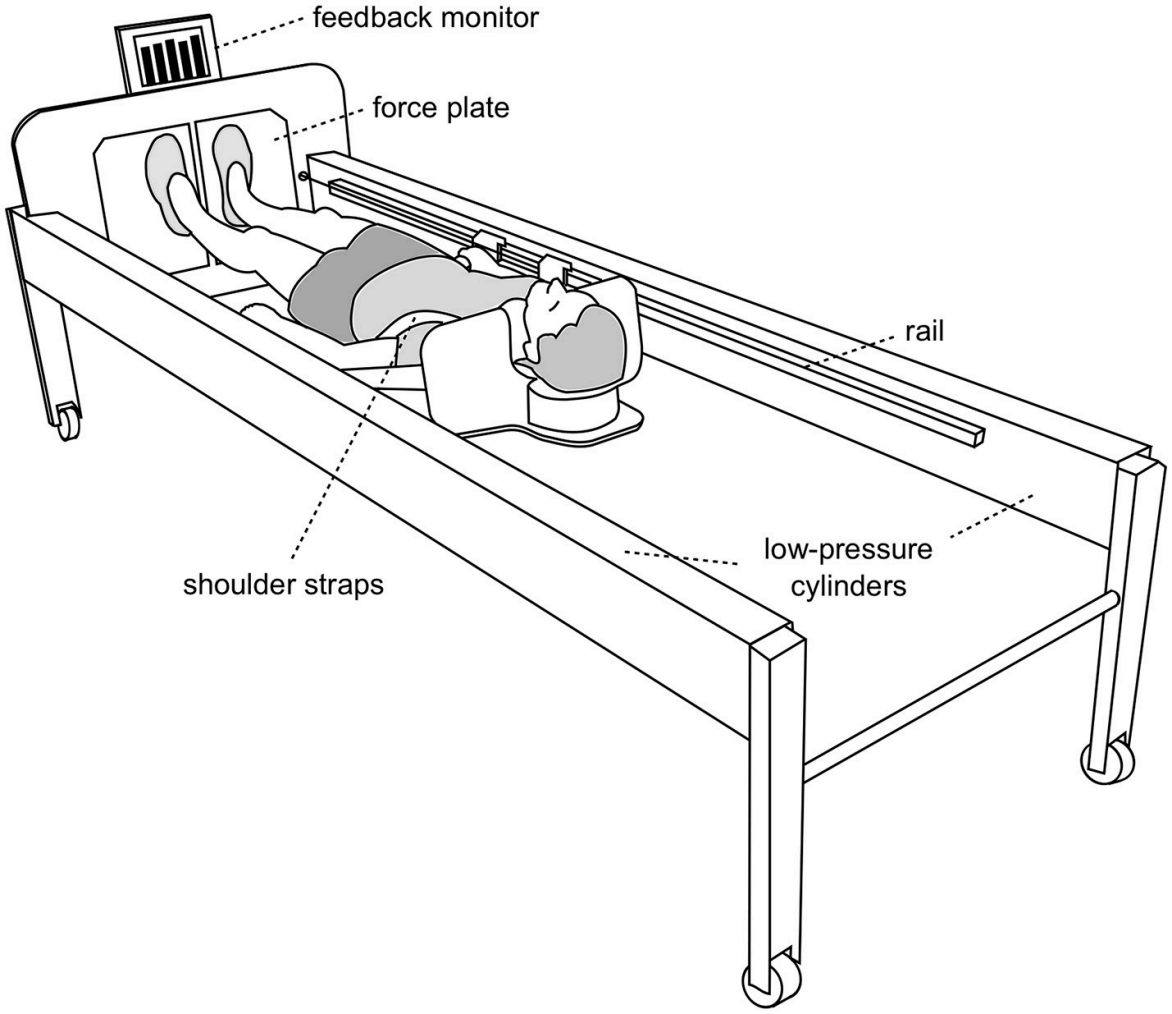
The training device (sledge jump system, SJS). The participant is fixed to the wooden sledge with shoulder straps, and his thighs rest on additional straps. The straps are attached to the rails and can slide in the direction of the rails with minimal friction. The forces generated by the two low-pressure cylinders substitute the gravitational force. Any force between zero and 1800 N can be set by altering the pressure of the cylinders. The participant stands on two force plates (separated, one for each foot). The figure was first published in Kramer et al. (2017b).

The sledge jump system (SJS, see Figure 2) was developed by Novotec Medical GmbH (Pforzheim, Germany). In brief, the subject can jump in the system with hardly any restrictions concerning the joint movements, allowing almost natural jumps (Kramer et al., 2010, 2012a) with different acceleration levels (Kramer et al., 2012b). Since the movement direction is along a horizontal axis, the forces generated by the two low-pressure cylinders substitute the gravitational force. One cylinder can generate 450 N at full capacity, i.e., any force between zero and 1800 N can be set by altering the pressure of the cylinders. Ground reaction forces (GRF) were recorded via two force plates, and the position of the sledge as recorded via an incremental encoder. During nine 30-min sessions during BDC, all subjects were familiarized with the correct jumping technique in the SJS. Each familiarization session consisted of a warm-up (see training) and then six countermovement jumps (CMJ) and four series of 10 hops each. The force in the SJS was gradually increased from 50% body weight in the first session up to 100% body weight in the last session. Participants were shown the correct jumping technique and received verbal feedback about their technique after each series of jumps. In addition, visual feedback about the target parameters (peak force for the hops and jump height for the CMJs) was provided for each jump on the SJS feedback monitor.

### Training

The training protocol for the JUMP group during the 60 days of HDT comprised a total of 48 training sessions. On average, each session consisted of 4 × 12 countermovement jumps and 2 × 15 repetitive hops, preceded by a warm-up that consisted of 6 squats and heel raises, 3 submaximal countermovement jumps and 1 × 10 submaximal repetitive hops. All sessions were supervised and documented, and the average time spent exercising during one session was ~3 min. Peak forces during the training amounted to 3.6 ± 0.4 kN and peak power to 3.4 ± 0.3 kW. Further details about the training can be found in Kramer et al. (2017b).

### Jump tests

Each test consisted of jumps on a force plate (Leonardo GRFP, Novotec medical GmbH, Pforzheim, Germany)—two CMJs, one squat jump and two series of 10 bilateral hops—and jumps in the training device (two CMJs, two series of 10 hops), in counterbalanced order. Prior to the first test, all participants were shown and practiced the correct execution of all jumps: hands were placed on the hips—or held on to the straps in the SJS—and subjects were instructed to jump with maximal effort. For the CMJs, the instruction was to “Quickly drop to a squat and then immediately jump as high as possible,” for the hops it was “Jump as stiff as possible, i.e., flex the ankle, knee and hip joint as little as possible while still jumping as high as the high stiffness allows; do not let the heels touch the plate during landing, keep the contact time as short as possible and jump as constant as possible.” The squat jumps were always performed with an initial knee angle of 90° with the instruction to “Jump as high as possible without countermovement prior to the jump.” After warm-up—consisting of 10 s of tapping, 10 submaximal hops, and three submaximal countermovement jumps–the jumps were performed in counterbalanced order, with a break of 1 min between each jump or series of hops. The GRF perpendicular to the force plates were recorded separately for the right and the left foot and synchronized to the EMG signals via a data acquisition unit (Power1401-3, CED, Cambridge, United Kingdom).

### Electromyography

Wireless surface electrodes (Trigno, Delsys, USA) were placed over M. soleus (SOL), M. gastrocnemius medialis (GM), M. tibialis anterior (TA), M. rectus femoris (RF), M. vastus lateralis (VL), and M. biceps femoris (BF) of the left leg. The longitudinal axes of the electrodes were in line with the presumed direction of the underlying muscle fibers. Interelectrode resistance was reduced by means of shaving and light abrasion of the skin. The EMG signals were wirelessly transmitted to the base station (band-pass filter 20–450 Hz, effective signal gain of 909) and sampled with 2 kHz using the data acquisition unit (Power1401-3, CED, Cambridge, United Kingdom).

### Data availability

The data that support the findings of this study are available from the authors upon reasonable request. The data are to be made available in the Erasmus Experiment Archive (http://eea.spaceflight.esa.int/portal/).

### Data processing

For the CMJs and SJs on the ground, the jump height was calculated based on the velocity at takeoff [antiderivative of the GRF; jump height = (velocity at takeoff)^2^/2 g], and power was calculated as the product of GRF and velocity. The higher one of the two CMJs was included in the further analyses. The jump height and power of the CMJs in the SJS was calculated similarly, except that the velocity was determined as the derivative of the position signal acquired by the incremental encoder. For the repetitive hops on the ground and in the SJS, the first two hops of each series as well as hops performed with heel contact were discarded; peak force, rate of force development and ground contact time for each of the remaining hops in the two series of 10 hops was determined and then averaged. Rate of force development was calculated as the peak force divided by the time from touchdown until the force signal reached its peak.

**Figure 3:**
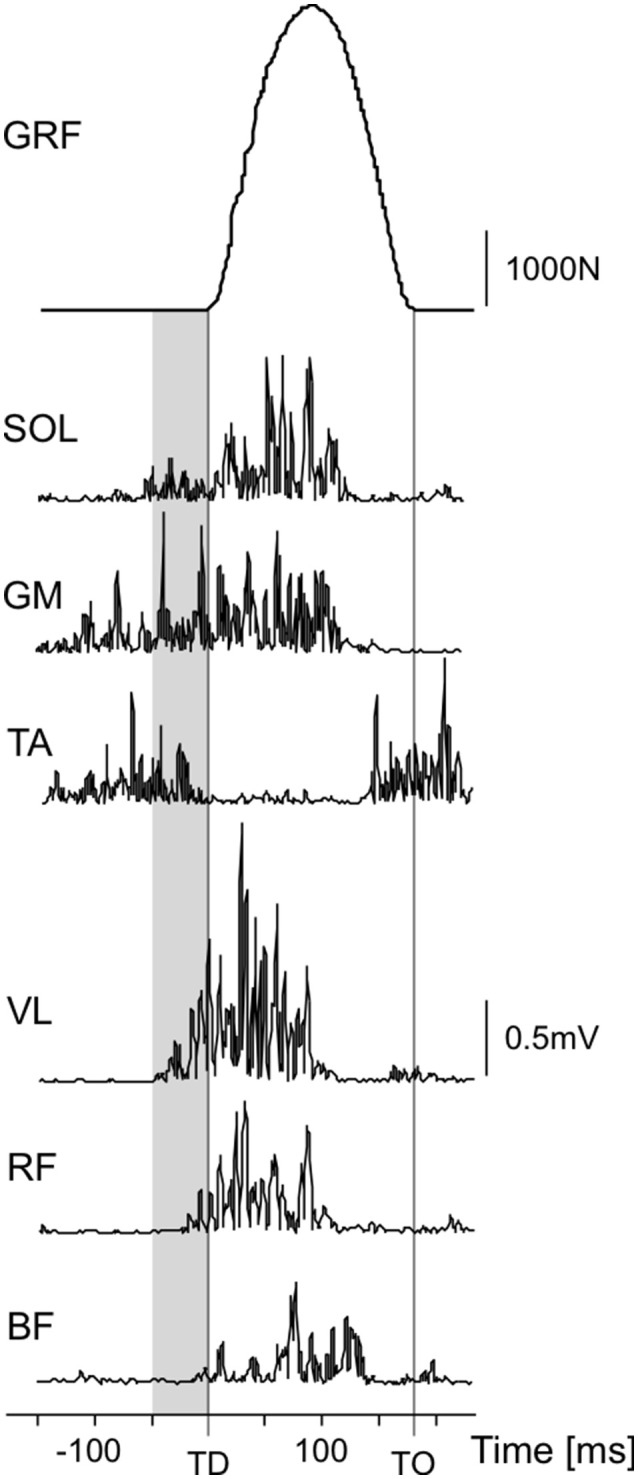
Exemplary data of one reactive hop: ground reaction force (GRF) and rectified EMG signals of soleus (SOL), gastrocnemius medialis (GM), tibialis anterior (TA), vastus lateralis (VL), rectus femoris (RF), and biceps femoris (BF). The preactivity phase (50 ms before touchdown (TD) until touchdown) is marked in gray, the take-off (TO) is marked with a vertical line.

After removing DC offsets, the EMG signals were rectified. Then, the mean amplitude voltage (MAV) was calculated for every jump, in case of the hops for the preactivity phase (50 ms before touchdown until touchdown, see Figure 3), and in case of the countermovement jumps and squat jumps for the concentric phase of the jumps (low point of the jump, velocity = 0, until takeoff). Just like for force and power, the MAV was then averaged for all valid hops of the 2 × 10 hops, and for the CMJs only the MAV of the highest jump was further analyzed.

### Statistics

After verifying distribution normality with Kolmogorov-Smirnov tests, changes in response to bed rest were assessed with repeated measures analyses of variance (ANOVA), using time (BDC-1 and R+0) as repeated measure and group (JUMP, CTRL) as inter-subject factor. In case Mauchly's test of sphericity produced significant results, the Greenhouse-Geisser correction was applied. Alpha level was set to 0.05 when testing for statistical differences. To test agreement with baseline values, non-inferiority statistics were used (Walker and Nowacki, 2011): the 90% confidence intervals were calculated for the differences to baseline values (BDC-1). The acceptable bounds were determined for each parameter separately, based on the differences observed between single jumps during BDC-1 (Kramer et al., 2010). If the results were statistically non-inferior to baseline (i.e., if the confidence interval lay above the lower bound), the respective parameter is marked with a ≥ symbol. Effect sizes for interaction effects for the performance data were calculated via Hedge's g (mean pre-post difference in the control group minus mean pre-post difference in the training group, divided by the pooled standard deviation). Sample size estimations were based on the results of previous bed rest studies, with an additional margin for potential dropouts (power of 0.9, alpha of 0.05, effect size of 0.4). The analyses were executed with SPSS 21.0 (SPSS, Inc., Chicago, IL). Group data are presented as means ± standard deviations (SD).

## Results

**Figure 4:**
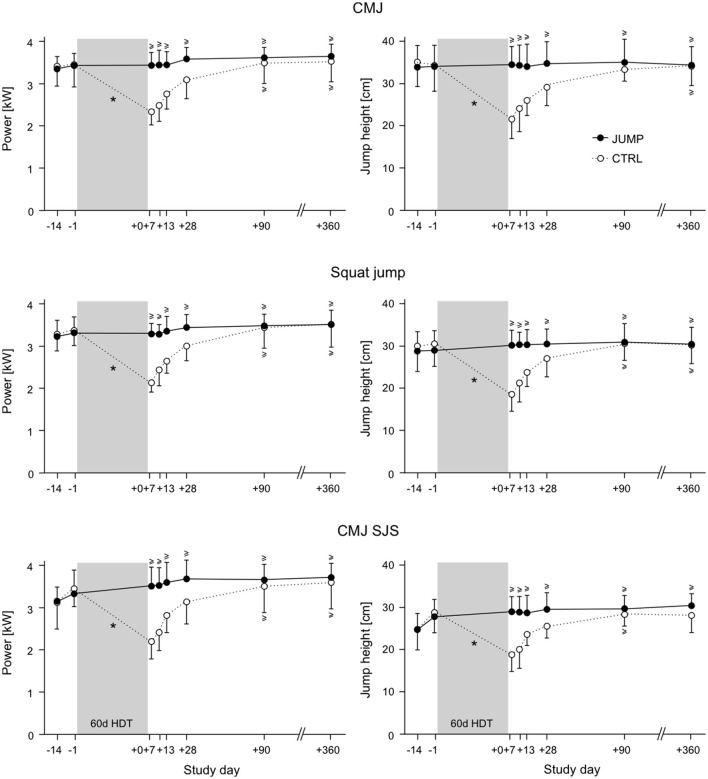
Means and standard deviations for power and jump height of all participants in the training group (JUMP, full circles) and the control group (CTRL, open circles). CMJ: countermovement jump, CMJ SJS: countermovement jump in the sledge jump system. The bed rest phase is marked in gray. The timeline starts at the beginning of the ambulatory phase (BDC-14) and ends with the last follow-up measurement (R+360, one year after the end of the bed rest phase). A ^*^ symbol denotes a statistically significant group × time interaction effect for the BDC-1 and the R+0 measurements, and a ≥ symbol denotes statistical non-inferiority to baseline.

**Figure 5:**
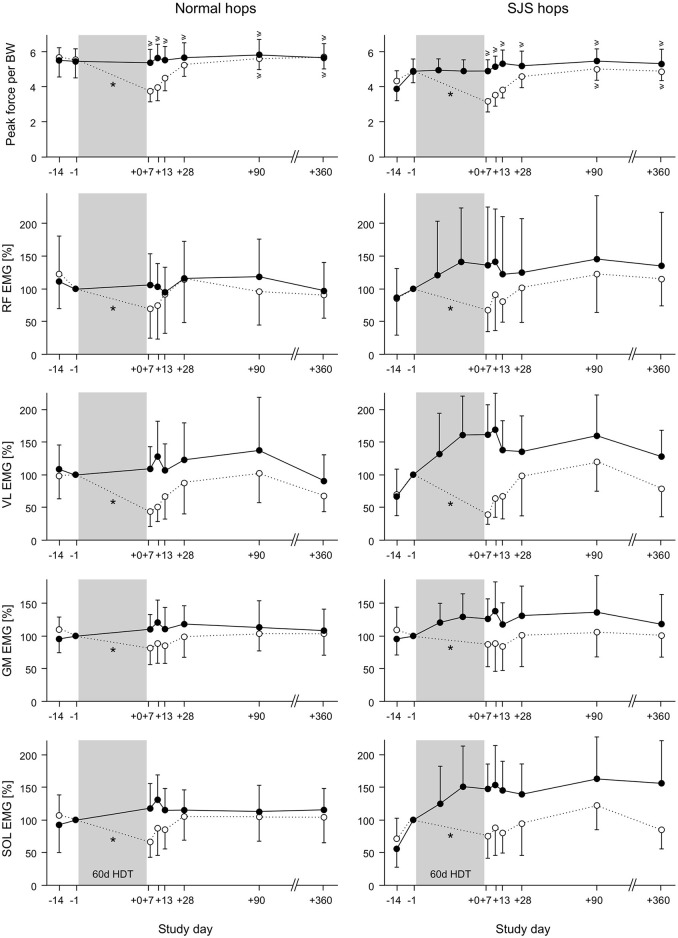
Means and standard deviations for peak force normalized to body weight and muscle activity (mean amplitude voltage during preactivity, i.e., the 50 ms before ground contact) of all participants in the training group (JUMP, full circles) and the control group (CTRL, open circles). The EMG data were normalized to the values directly before bed rest (BDC-1). Left: repetitive hops on the ground; right: repetitive hops in the sledge jump system (SJS). For the training group, two additional measurements were taken during two training sessions in the bed rest phase (HDT20 and HDT40). The bed rest phase is marked in gray. A ^*^ symbol denotes a statistically significant group × time interaction effect for the BDC-1 and the R+0 measurements, and a ≥ symbol denotes statistical non-inferiority to baseline (performance measurements only).

In the training group, all of the measured jump performance parameters remained constant or increased after the 60 days of bed rest, whereas in the control group they showed decreases between 30 and 60%, see Table 1. This was true for all three types of jumps (CMJ, SJ, repetitive hops), both on the ground and in the SJS (see Figures 4, 5). The ANOVA comparing the values directly before bed rest (BDC-1) to the values directly after bed rest (R+0) all showed significant group^*^time interaction effects, with large between-group effect sizes (see Table 1). Non-inferiority statistics showed that the performance parameters did not differ significantly between R+0 and BDC-1 in the training group, and that the pronounced decreases observed in the control group were mostly recovered 3 months after the end of bed rest.

**Table 1:** Jump performance.

	**JUMP BDC-1**	**JUMP R+0**	**CTRL BDC-1**	**CTRL R+0**	Interaction group*time	**Hedge's g**
CMJ power abs. [kW]	3.4 ± 0.3	3.4 ± 0.3 ≥+0 ± 10%	3.4 ± 0.5	2.4 ± 0.3 −31 ± 8%	*F*_1, 21_ = 47.5 *p* < 0.001	3.42
CMJ power rel. [W/kg]	44 ± 5	45 ± 5≥+2 ± 9%	45 ± 5	32 ± 5 −28 ± 8%	*F*_1, 21_ = 61.6 *p* < 0.001	3.50
CMJ height [cm]	34 ± 5	34 ± 4 ≥+1 ± 6%	34 ± 6	22 ± 5 36 ± 12%	*F*_1, 21_ = 68.3 *p* < 0.001	3.87
SJ power abs. [kW]	3.3 ± 0.3	3.3 ± 0.2 ≥+1 ± 7%	3.4 ± 5	2.1 ± 0.2 −35 ± 13%	*F*_1, 21_ = 41.5 *p* < 0.001	3.53
SJ power rel. [W/kg]	42 ± 6	43 ± 4 ≥+3 ± 7%	44 ± 6	29 ± 4 −33 ± 13%	*F*_1, 21_ = 47.4 *p* < 0.001	3.42
SJ height [cm]	29 ± 4	30 ± 4 ≥+4 ± 9%	31 ± 5	19 ± 4 −37 ± 18%	*F*_1, 21_ = 37.4 *p* < 0.001	2.89
CMJ SJS power abs. [kW]	3.4 ± 0.3	3.5 ± 0.4 ≥+5 ± 8%	3.5 ± 0.5	2.2 ± 0.4 −36 ± 9%	*F*_1, 21_ = 86.2 *p* < 0.001	4.76
CMJ SJS power rel. [W/kg]	44 ± 5	47 ± 5 ≥+8 ± 8%	46 ± 4	31 ± 5 −33 ± 10%	*F*_1, 21_ = 102.8 *p* < 0.001	4.53
CMJ SJS height [cm]	28 ± 4	29 ± 4 ≥+4 ± 10%	29 ± 3	19 ± 4 −34 ± 15%	*F*_1, 21_ = 44.7 *p* < 0.001	3.10
Hop force abs. [kN]	4.2 ± 0.7	4.0 ± 0.5 ≥−2 ± 9%	4.1 ± 0.4	2.7 ± 0.5 −35 ± 7%	*F*_1, 21_ = 64.2 *p* < 0.001	3.85
Hop force rel. to BW [a.u.]	5.5 ± 1.0	5.4 ± 0.7 ≥−1 ± 9%	5.5 ± 0.6	3.7 ± 0.6 −32 ± 8%	*F*_1, 21_ = 63.6 *p* < 0.001	3.87
Hop GCT [ms]	178 ± 18	179 ± 18 ≥+0 ± 5%	171 ± 20	219 ± 37 +28 ± 18%	*F*_1, 21_ = 35.4 *p* < 0.001	−2.16
Hop RFD [kN/s]	53 ± 16	53 ± 13 ≥+4 ± 21%	55 ± 10	26 ± 9 −53 ± 14%	*F*_1, 21_ = 40.3 *p* < 0.001	3.15
Hop SJS force abs. [kN]	3.7 ± 0.5	3.7 ± 0.4 ≥−1 ± 9%	3.7 ± 0.5	2.3 ± 0.4 −40 ± 7%	*F*_1, 21_ = 87.2 *p* < 0.001	4.97
Hop SJS force rel. to BW []	4.9 ± 0.7	4.9 ± 0.6 ≥+1 ± 9%	4.9 ± 0.6	3.2 ± 0.7 −38 ± 6%	*F*_1, 21_ = 154.3 *p* < 0.001	5.13
Hop SJS GCT [ms]	172 ± 25	166 ± 24 ≥−3 ± 7%	176 ± 21	250 ± 47 +48 ± 18%	*F*_1, 21_ = 61.2 *p* < 0.001	−3.81
Hop SJS RFD [kN/s]	55 ± 15	56 ± 14 ≥+4 ± 23%	55 ± 15	22 ± 11 −64 ± 11%	*F*_1, 21_ = 49.4 *p* < 0.001	3.74

*Means and standard deviations of the jump performance tests, separately for the training group (JUMP) and the control group (CTRL), once directly before (BDC-1) and once directly after bed rest (R+0). Five tests were conducted: countermovement jumps (CMJ) and squat jumps (SJ) on a force plate, countermovement jumps in the training device (sledge jump system, SJS), and repetitive hops both on a force plate and in the training device. The percent values reflect the changes observed at R+0 compared to baseline (BDC-1). BW, body weight; GCT, ground contact time; RFD, rate of force development. If the R+0 value is statistically non-inferior compared to baseline, it is marked with a ≥ symbol. Hedge's g refers to the mean changes in the training group vs. the mean changes in the control group*.

The results of the muscle activity analyses were in line with the changes observed for the performance parameters, i.e., no changes or increases in the training group and decreases in the control group, with the most pronounced time ^*^ group interaction effects in m. vastus lateralis, see Figure 5 and Table 2.

**Table 2:** EMG data.

	**JUMP BDC-1**	**JUMP R+0**	**CTRL BDC-1**	**CTRL R+0**	Interaction group*time
CMJ RF [mV]	0.15 ± 0.07	0.13 ± 0.05≥	0.15 ± 0.06	0.12 ± 0.05	*F*_1, 21_ = 0.4 *p* = 0.52
CMJ VL [mV]	0.23 ± 0.13	0.26 ± 0.12≥	0.26 ± 0.11	0.14 ± 0.07	*F*_1, 21_ = 30.4 ***p***<**0.001**
CMJ BF [mV]	0.05 ± 0.03	0.05 ± 0.02≥	0.04 ± 0.01	0.04 ± 0.03≥	*F*_1, 21_ = 0.4 *p* = 0.55
CMJ GM [mV]	0.11 ± 0.04	0.12 ± 0.05≥	0.13 ± 0.03	0.09 ± 0.03	*F*_1, 21_ = 6.4 ***p*** = **0.02**
CMJ SOL [mV]	0.12 ± 0.03	0.11 ± 0.02≥	0.13 ± 0.02	0.11 ± 0.05	*F*_1, 21_ = 0.3 *p* = 0.60
CMJ TA [mV]	0.05 ± 0.03	0.04 ± 0.02≥	0.03 ± 0.01	0.03 ± 0.02≥	*F*_1, 21_ = 0.04 *p* = 0.85
SJ RF [mV]	0.14 ± 0.06	0.13 ± 0.05≥	0.17 ± 0.10	0.11 ± 0.05	*F*_1, 21_ = 6.5 ***p*** = **0.02**
SJ VL [mV]	0.21 ± 0.14	0.25 ± 0.11≥	0.26 ± 0.09	0.14 ± 0.08	*F*_1, 21_ = 17.6 ***p***<**0.001**
SJ BF [mV]	0.04 ± 0.01	0.05 ± 0.02≥	0.03 ± 0.01	0.03 ± 0.01≥	*F*_1, 21_ = 4.0 *p* = 0.06
SJ GM [mV]	0.10 ± 0.03	0.11 ± 0.05≥	0.13 ± 0.05	0.08 ± 0.03	*F*_1, 21_ = 12.4 ***p*** = **0.002**
SJ SOL [mV]	0.12 ± 0.04	0.12 ± 0.02≥	0.12 ± 0.03	0.10 ± 0.04	*F*_1, 21_ = 1.1 *p* = 0.30
SJ TA [mV]	0.04 ± 0.01	0.03 ± 0.01≥	0.04 ± 0.02	0.03 ± 0.02≥	*F*_1, 21_ = 0.1 *p* = 0.75
CMJ_SJS_ RF [mV]	0.12 ± 0.09	0.12 ± 0.05≥	0.13 ± 0.04	0.10 ± 0.07	*F*_1, 21_ = 2.8 *p* = 0.11
CMJ_SJS_ VL [mV]	0.21 ± 0.17	0.25 ± 0.17≥	0.22 ± 0.10	0.12 ± 0.06	*F*_1, 21_ = 17.7 ***p***<**0.001**
CMJ_SJS_ BF [mV]	0.03 ± 0.02	0.05 ± 0.02≥	0.03 ± 0.02	0.03 ± 0.01≥	*F*_1, 21_ = 12.1 ***p*** = **0.002**
CMJ_SJS_ GM [mV]	0.11 ± 0.04	0.11 ± 0.06≥	0.13 ± 0.03	0.10 ± 0.03	*F*_1, 21_ = 2.3 *p* = 0.14
CMJ_SJS_ SOL [mV]	0.12 ± 0.05	0.11 ± 0.05≥	0.11 ± 0.03	0.09 ± 0.04	*F*_1, 21_ = 0.1 *p* = 0.74
CMJ_SJS_ TA [mV]	0.03 ± 0.01	0.03 ± 0.02≥	0.03 ± 0.01	0.04 ± 0.01≥	*F*_1, 21_ = 2.9 *p* = 0.10
Hop RF [mV]	0.05 ± 0.02	0.05 ± 0.03≥	0.07 ± 0.05	0.04 ± 0.01	*F*_1, 21_ = 6.8 ***p*** = **0.02**
Hop VL [mV]	0.11 ± 0.06	0.12 ± 0.09≥	0.14 ± 0.10	0.06 ± 0.04	*F*_1, 21_ = 12.7 ***p*** = **0.002**
Hop BF [mV]	0.03 ± 0.02	0.03 ± 0.01≥	0.05 ± 0.03	0.03 ± 0.02	*F*_1, 21_ = 8.1 ***p*** = **0.01**
Hop GM [mV]	0.12 ± 0.04	0.12 ± 0.02≥	0.14 ± 0.08	0.10 ± 0.03	*F*_1, 21_ = 4.9 ***p*** = **0.04**
Hop SOL [mV]	0.06 ± 0.03	0.07 ± 0.04≥	0.08 ± 0.03	0.05 ± 0.02	*F*_1, 21_ = 12.8 ***p*** = **0.002**
Hop TA [mV]	0.05 ± 0.02	0.04 ± 0.02≥	0.05 ± 0.03	0.02 ± 0.01	*F*_1, 21_ = 0.9 *p* = 0.36
Hop_SJS_ RF [mV]	0.07 ± 0.03	0.07 ± 0.03≥	0.08 ± 0.06	0.05 ± 0.04	*F*_1, 21_ = 6.3 ***p*** = **0.02**
Hop_SJS_ VL [mV]	0.10 ± 0.05	0.16 ± 0.08≥	0.14 ± 0.11	0.06 ± 0.05	*F*_1, 21_ = 22.6 ***p***<**0.001**
Hop_SJS_ BF [mV]	0.04 ± 0.03	0.04 ± 0.01≥	0.04 ± 0.04	0.03 ± 0.02≥	*F*_1, 21_ = 1.9 *p* = 0.2
Hop_SJS_ GM [mV]	0.10 ± 0.03	0.12 ± 0.04≥	0.13 ± 0.08	0.10 ± 0.02	*F*_1, 21_ = 8.8 ***p*** = **0.01**
Hop_SJS_ SOL [mV]	0.06 ± 0.03	0.09 ± 0.03≥	0.08 ± 0.03	0.07 ± 0.03	*F*_1, 21_ = 14.0 ***p*** = **0.002**
Hop_SJS_ TA [mV]	0.05 ± 0.03	0.04 ± 0.02≥	0.05 ± 0.02	0.02 ± 0.01	*F*_1, 21_ = 2.1 *p* = 0.17

*Means and standard deviations of the muscle activity during the jumps (CMJ, countermovement jump; SJ, squat jump; CMJ_SJS_, countermovement jump in the sledge jump system; Hop, reactive hops; Hop_SJS_, reactive hops in the sledge jump system) for the training group (JUMP) and the control group (CTRL), once directly before (BDC-1) and once directly after bed rest (R+0, percent values compared to BDC-1). For the CMJ and the SJ the values refer to the mean amplitude voltage during the concentric phase of the jump, for the hops they refer to the preactivity during the 50 ms before tochdown. If the R+0 value of the training group is statistically non-inferior to baseline, it is marked with a ≥ symbol*.

*Bold values indicate significant values (p < 0.05)*.

## Discussion

The jump training was able to maintain peak power, peak force and rate of force development of the lower limb muscles after 2 months of physical inactivity during bed rest, and this effect was not limited to the training device. In the control group, the pronounced decrease in performance due to physical inactivity slowly recovered in the weeks following the end of bed rest and had returned to baseline values after ~3 months.

Previous bed rest studies with different types of countermeasures have shown that peak power is hard to maintain over prolonged periods of physical inactivity, and that peak power as well as jump height can decrease even if the countermeasure is able to maintain maximal force during isometric contractions. For example, nutritional interventions such as protein supplementation were not successful at all (Trappe et al., 2007), and the otherwise quite effective countermeasure used in a previous study with similar duration—a combination of resistive exercise and whole body vibration—still resulted in a decrease in the training group's power and jump height of 12 and 14%, respectively (Buehring et al., 2011). In fact, the jump training used in the present study is the first countermeasure that maintained peak power and rate of force development during jumps after long-term bed rest. This success is most probably due to the nature of the training, as it consisted only of countermovement jumps and reactive hops, i.e., movements with very high power and rate of force development. It underpins the importance of including high-power movements into training programs designed to prevent the detrimental effects of inactivity.

The training effect was not restricted to jumps in the training device, but transferred to “natural” jumps on the ground. Even performance in the squat jump, which does not rely on the stretch-shortening cycle and was not part of the training program, was also maintained in the training group. This is not a given, as training effects can be quite task- and device-specific (Giboin et al., 2015). For instance, the concentric-eccentric resistance training device used during a 90-day bed rest study (flywheel) was successful in maintaining device-specific peak power, but this did not transfer to other tasks (Alkner and Tesch, 2004). Possibly, the advantage of the jump training used in the present study was that jumps are a whole-body movement, which have been shown to elicit larger performance improvements than isolated single-joint movements, especially regarding lower limb power (Blackburn and Morrissey, 1998; Stone et al., 2000). Another contributing factor may have been that the training consisted only of jumps, i.e., an exercise mode where each repetition requires maximal effort. There is evidence that maximal effort—either achieved by loads near the one-repetition maximum or by high actual or intended velocity—is an important factor in strength and power training (Behm and Sale, 1993; Fry, 2004).

One could argue that it is not very surprising that a jump training maintained jump performance, whether this is in the training system or on the ground, but the training was also very effective in maintaining isometric MVC, muscle mass, and even aerobic capacity, although these parameters decreased by up to 40% in the control group (Kramer et al., 2017a,b). Thus, with adequate programming, a high-power training is also effective for strength and can act as a type of high-intensity interval training for cardiovascular and aerobic effects.

The muscle activity during the jump performance tests corresponded to the performance changes observed: there was no change or even an increase in the training group, whereas the control group exhibited a marked decrease in muscle activity in the leg extensors, most pronounced in vastus lateralis and the gastrocnemius medialis. These changes in muscle activity were somewhat task-specific: for the hops all leg extensors were affected, whereas for the countermovement jumps and the squat jumps, the two groups differed mainly for the extensors of the thigh, possibly due to the greater contribution of the thigh muscles during CMJ and SJ (Fukashiro and Komi, 1987). In the training group, the increase in muscle activity was most pronounced for the repetitive hops in the training device, pointing toward a task- and device-specific adaptation. The progress during the bed rest phase (compare Figure 5) suggests that despite the nine familiarization sessions before the start of the bed rest phase, this adaptation was still ongoing after several weeks of training during the bed rest phase. The effect of the familiarization—in particular for the repetitive hops—is apparent in the clear increase in performance and muscle-specific increase of the preactivity when comparing the BDC-14 to the BDC-1 values, i.e., at the start of the 2-week ambulatory phase before the nine familiarization sessions and just before bed rest, after the familiarization. It is interesting to note that in the training group, these adaptations partly persisted during the follow-up measurements, just like the increase in performance during the hops in the SJS.

### Recovery of the effects of physical inactivity

The analysis of the follow-up performance measurements during the recovery period showed that even though the decrease in power and rate of force development was quite high in the control group, this performance was reversible and was recovered after about 3 months. This is faster than previously reported recovery times of 5 months (Rittweger et al., 2007), but in that study bed rest duration was 90 days instead of 60 days, suggesting that the recovery time depends on the bed rest duration.

The control group's recovery of the muscle activity during the jumps was mostly completed after 1 month, i.e., somewhat faster than the recovery of jump performance, pointing toward a relatively fast recovery of inter- and intramuscular coordination and a slightly slower recovery of muscle mass and other structural changes.

## Limitations

It is important to remember that the muscle activity data were not normalized. This means that in addition to the changes in muscle activation in response to physical activity and jump training, the EMG was potentially influenced for instance by a bed-rest induced fluid shift, slight differences in electrode placement, changes in skin impedance and changes in lean or fat mass. However, most of these potential confounding factors would have occurred in both groups, thus hardly influencing the group × time interaction effects of interest. We chose not to normalize the EMG data (for example to the EMG during MVC) because this would have masked most group differences: the training group maintained their MVC after bed rest, whereas the control group lost up to 40% (Kramer et al., 2017a). Thus, normalizing the EMG data of the jumps to the EMG activity during MVC—which was also maintained in the training group and reduced in the control group—would eradicate the differences between the groups. Therefore, not normalizing EMG data when groups are hardly comparable—for instance patients vs. healthy populations—has been suggested to facilitate adequate interpretation of the data (Cholewicki et al., 2011).

## Conclusion

The short jump training successfully maintained the neuromuscular system's capability to perform complex movements with high power output, high peak forces, high RFD and the necessary muscle activation pattern, whereas the control group exhibited a marked decline. This finding highlights the effectiveness of including whole-body exercises requiring high power and rate of force development into training programs aimed at maintaining the full capabilities of the neuromuscular system.

## Author contributions

AK contributed to study preparation, data acquisition, data analysis, figure preparation, and manuscript draft. JK contributed to study preparation, data acquisition, and manuscript revision. RR contributed to data acquisition and manuscript revision. MG, AG, GA, DB, and DF contributed to study preparation and manuscript revision. All authors approved the final manuscript and are accountable for all aspects of the work.

### Conflict of interest statement

The authors declare that the research was conducted in the absence of any commercial or financial relationships that could be construed as a potential conflict of interest.
